# Automated Detection of Leakage in Fluorescein Angiography Images with Application to Malarial Retinopathy

**DOI:** 10.1038/srep10425

**Published:** 2015-06-01

**Authors:** Yitian Zhao, Ian J. C. MacCormick, David G. Parry, Sophie Leach, Nicholas A. V. Beare, Simon P. Harding, Yalin Zheng

**Affiliations:** 1 School of Mechanical Engineering, Beijing Institute of Technology, Beijing, China; 2 Department of Eye and Vision Science, University of Liverpool, Liverpool, United Kingdom; 3 Malawi-Liverpool-Wellcome Trust Clinical Research Programme, Blantyre, Malawi; 4 St Paul’s Eye Unit, Royal Liverpool University Hospital, Liverpool, United Kingdom

## Abstract

The detection and assessment of leakage in retinal fluorescein angiogram images is important for the management of a wide range of retinal diseases. We have developed a framework that can automatically detect three types of leakage (large focal, punctate focal, and vessel segment leakage) and validated it on images from patients with malarial retinopathy. This framework comprises three steps: vessel segmentation, saliency feature generation and leakage detection. We tested the effectiveness of this framework by applying it to images from 20 patients with large focal leak, 10 patients with punctate focal leak, and 5,846 vessel segments from 10 patients with vessel leakage. The sensitivity in detecting large focal, punctate focal and vessel segment leakage are 95%, 82% and 81%, respectively, when compared to manual annotation by expert human observers. Our framework has the potential to become a powerful new tool for studying malarial retinopathy, and other conditions involving retinal leakage.

Fluorescein angiography (FA) is a type of imaging commonly used in ophthalmology clinics that provides a map of retinal vascular structure and function by highlighting blockage of, and leakage from, retinal vessels^1^. The value of FA in differential diagnosis of retinal diseases, such as age-related macular degeneration (AMD) and diabetic retinopathy (DR), is well recognised. Malarial retinopathy (MR) is made up of a collection of important signs in paediatric cerebral malaria (CM). MR reveals almost all the different types of angiographic vascular abnormalities that are common to other retinal conditions, and as such it is convenient to use this condition to develop semi-automatic or fully automatic tools for quantitatively assessing the retinal abnormalities. On the other hand, similarities between eye and brain relevant to cerebral malaria seems to suggest that the retina may be a good source of potential biomarkers which might cast light on cerebral malaria disease processes^2^. However, with few exceptions^3^,^4^ existing descriptions of paediatric MR are qualitative or semi-quantitative, and based on ophthalmoscopic examination rather than more sophisticated imaging techniques. FA captures a range of retinal abnormalities in paediatric MR, in addition to those visible with ophthalmoscop^5^. These include capillary non-perfusion^3^, intravascular filling defects, and several types of leakage. Retinal vessel leakage is particularly relevant to cerebral malaria, since the blood-retinal barrier is similar to the blood-brain barrier^6^, and leakage from the latter could contribute to the brain swelling commonly seen in paediatric cerebral malaria^7^.

Three types of leakage can be observed in malarial retinopathy: large focal, punctate focal, and vessel segment leakage (Fig. 1). Large focal leakage describes one or more large, usually circular areas of leak, where the greatest linear diameter is larger than 125 *μ*m. Large focal leak is characteristically associated with haemorrhage^8^. Punctate focal leak involves small but intensely bright sites of leak, where the greatest linear diameter is less than 125 *μ*m. Leakage from vessel segments appears as increased brightness and blurring of vessels. In malarial retinopathy this leakage appears to almost exclusively affect venules, and spare arterioles. Arterioles, which lie immediately adjacent to retinal venules, therefore provide a useful reference against which venule brightness and blur can be compared. Vessel leakage is associated with capillary non-perfusion and retinal whitening^8^.

To the best of our knowledge, there is no automated method to detect leakage in MR. Leakage detection in other ocular diseases has received little, if any, attention. Phillips *et al.* used unsupervised image processing and gradient thresholding with region growing^9^. But this method was only applied to six cases. Efforts to detect choroidal neovascularisation (CNV) seem to be relevant to detection of leakage in malarial retinopathy^10^,^11^, since CNV appear as focal hyperfluorescent areas on FA. However detection of CNV only involves analysis of a limited area of retina and does not evaluate the entire image. Trucco *et al.* used the evolution of pixel intensities to classify leakage regions in multiple frames with the AdaBoost classifier^12^,^13^. Tsai *et al.* also used the AdaBoost classifier and created severity maps of leakage regions^14^. However, supervised classifiers are limited by their requirement for training datasets derived from manual annotation. Manual annotation can be extremely time consuming, and the performance of the classifier is inherently dependent on the quality of this annotation.

In this paper we describe a novel unsupervised technique to quantify the type and severity of leakage in MR by a novel adaptation of the concept of salient features. When one sees the world, the brain relies on *attention* to capture the most salient details. *Selective attention* refers to the cognitive mechanism that determines which part of the plethora of sensory data is currently of most interest (i.e. *salient*)^15^. The cocktail party effect^16^ is a well-known example of selective attention. Visual saliency is of great importance in neurophysiology, psychology and computer vision. Visual saliency in 2-dimensional (2D) images is the perceptual quality that makes an object, person, or pixel stand out relative to its neighbours, and that captures one’s attention^17^. It indicates the relative importance of visual features and is closely related to characteristics of human perception and processing of visual stimuli17,18. Saliency originates from visual uniqueness, unpredictability, rarity, or surprise, and is often attributed to variations in image attributes like colour, gradient, edges, and boundaries^19^. Such attributes are characteristic of retinal leakage in FA images. For example, leakage of fluorescent dye causes a large difference in brightness between the leak and surrounding non-leaking areas. Therefore, the leaking regions may be defined as salient regions. We now describe our saliency computation, which is based on this rule: the salient region is always different from its surrounding context^20^.

We also evaluate the performance of our automated method against a human expert reference standard. By accurately quantifying neurovascular leakage, our method will facilitate analyses of associations between retinal vessel leakage and clinical outcome in cerebral malaria. It has similar potential for other systemic and ophthalmological conditions characterised by retinal leakage^1^.

## Results

The proposed detection framework described below (Methods) was applied to a collection of FA images with reference standard and the experiments provided promising results.

### Dataset

Our dataset consisted of retinal FA images taken from children with CM admitted to the Paediatric Research Ward, Queen Elizabeth Central Hospital, Blantyre, Malawi. All subjects had signs of MR on admission. 50-degree images were taken after pupil dilation with Tropicamide 1% and Phenylephrine 2.5%, using a Topcon 50-EX optical unit (Topcon, Japan) and Nikon E1-H digital camera. The size of all macula-centred images are 3,008 × 1,960. We created montages of several images to facilitate detection of large focal and punctate leakage in both macula and periphery, and to avoid over counting sites of leak. Note, because cerebral malaria patients are comatose the original images used in this paper were acquired from subjects lying in the left lateral position. Images used in this paper have been rotated to conform with ophthalmological convention. Image orientation does not affect our leakage detection method. Ethical approval for retinal examination and imaging was given by committees in Blantyre and at collaborating institutions. Consent was given by the parents/guardians of subjects before examination and imaging. The tenets of the Declaration of Helsinki were adhered to. The images were systematically sorted and graded for quality by the Reading Centre at St Paul’s Eye Unit, Royal Liverpool University Hospital and Department of Eye and Vision Science, University of Liverpool. Images used in our protocol were randomly chosen from patients with clinical outcomes of death, full recovery, and recovery with neurological complications.

We compared the results of our automated detection method against a reference standard of manual annotation by human experts. One grader (IJCM) defined the boundaries of each large focal leak, and the centre point of each punctate focal leak. Three graders used an in-house Matlab program version 2013a (Mathworks, Natick, CA) to label vessels as positive or negative for vessel segment leak. This program displayed the original image next to a copy where the centre of vessel segments was highlighted in yellow. Observers selected leaking vessels and non-leaking vessels in turn by clicking on the vessel segment of interest. The selected leaking vessels were then coloured red while non-leaking vessels were coloured green. In the first phase, a professional grader (SL) labelled the vessels independently. Her labels were combined with annotations from an ophthalmologist familiar with malarial retinopathy (IJCM), and a consensus between the two of them was used as the final reference standard. In order to assess inter-observer variation, a second professional grader (DGP) labelled the vessels using the same software and following the same guidelines in a masked pattern. All the observers were experienced in grading FA images of malarial retinopathy. Where human graders were uncertain whether vessel leakage was present or absent, vessels were left unlabelled and are not analysed in this study.

### Results of Large Focal Leakage Detection

**Figure 2:**
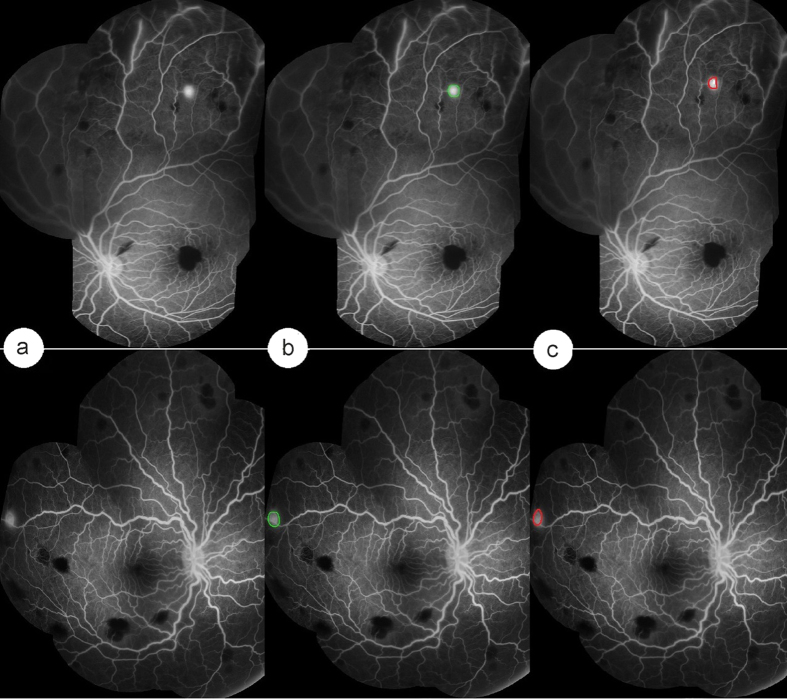
Detection of large focal leakage by our automated method and a human observer. (**a**) Input FA images with focal leakage. (**b**) Automated detection by our method. (**c**) Manual annotation.

We used 20 images (one per patient) with large focal leakage to evaluate detection of this leak type. In these images a total of 41 of sites of large focal leak were identified by the human expert reference standard. Detection sensitivity and the overlapping ratio (OR) are shown in Table 1. The OR is the ratio of pixels in regions detected by both computer and reference to pixels in areas annotated by human reference. The sensitivity of focal leakage detection is 0.95, which means our method only failed to detect 2 out of 41 focal leak sites, and has a false negative ratio of 0.1 per image. Meanwhile, there were no regions falsely identified as large focal leakage, and so our method has a false positive ratio of 0. The OR of focal leak area size is 0.89. Figure 2 illustrates two examples of large focal leakage detection by our method and manual annotation. The sizes of these foci estimated by a human grader (IJCM) and our framework are slightly different, but it is difficult to exactly define the boundary of a leaking area by hand since the contrast gradually fades at the edge of the lesion.

### Results of Punctate Focal Leakage Detection

**Figure 3:**
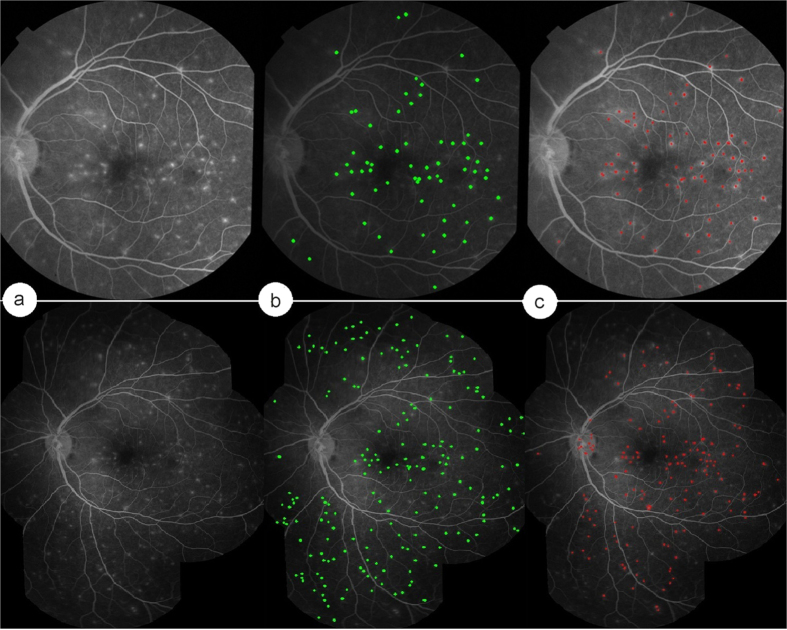
Detection of punctate focal leakage by our automated method and a human observer. (**a**) Input FA images with punctate leakage. (**b**) Automated detection by our method. (**c**) Manual annotations.

Punctate focal leakage is relatively less common compared with the other two leakage types (large focal and vessel segment leakage), only 6 patients were found with this leakage in database, and 10 images from these patients (including both macula-centred and montage images) were chosen for evaluation. According to the human reference standard there were 240 sites of punctate focal leakage. Table 1 demonstrates the evaluation results of our automated method to detect punctate focal leakage. The sensitivity of detection is 0.82. However, 26 sites were missed, leading to a false negative ratio of 2.6 per image. Moreover, 13 regions were falsely determined as punctate focal regions due to image artefact and imbalanced illumination. The false positive ratio is 1.3 per image. Figure 3 illustrates two examples of proposed detection results on punctate leakages and manual annotations. It shows most sites of punctate focal leak were detected by our automated framework.

### Results on Vessel Segment Leakage Detection

10 images (one per patient) with vessel segment leakage (overall 5,846 vessel segments) were used for the evaluation. According to the human reference standard these images contained 2,103 leaking vessel segments. Table 2 shows the evaluation results of our framework in terms of accuracy, sensitivity and specificity. It also shows a comparison between the human reference standard and annotation from a further human observer (DGP). Compared to the reference standard the specificity and accuracy of the human observer is only 0.736 and 0.758, which is 0.085 and 0.046 lower than our automated method, respectively. However, the sensitivity of second manual annotation is very similar to that of our automated method. We also considered the area under the curve (AUC), which is equal to 1 for a perfect system, as a single metric to quantify the performance of our framework in detecting vessel segments leakage. Our method is 0.05 higher than manual annotation. In brief, this table shows that our automated method to detect vessel segment leakage can perform as well or better than a single human expert, compared to a reference standard of dual human grading with consensus.

**Figure 4:**
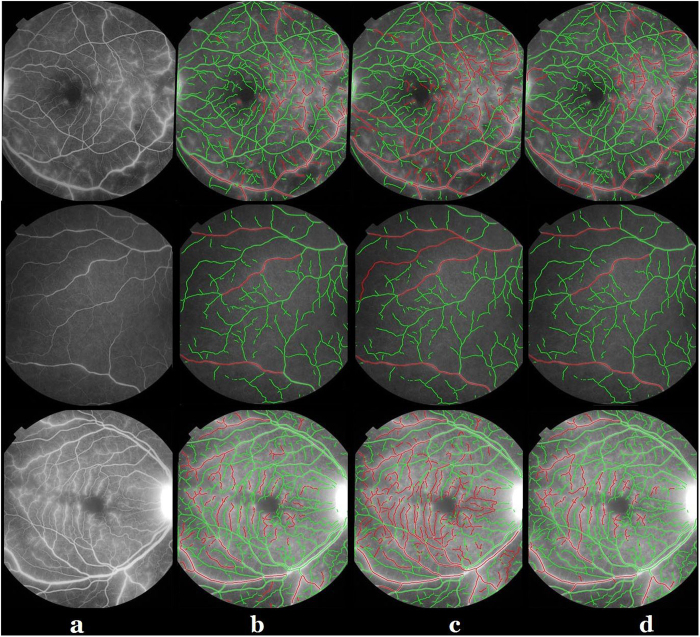
Vessel segment leakage detection by our automated method and human observers. (**a**) Example FA images. (**b**) Reference standard (consensus between experts SL and IJCM). (**c**) Manual annotations by a further human expert grader (DGP). (**d**) Leaking vessels detected by our automated method.

Figure 4 shows the results of our automated abnormal vessel detection on 3 example FA images. Normal vessels are shown in green while leaking vessels are shown in red. Figure 4d illustrates how our automated method classifies segmented vessels into normal and abnormal. Only vessels labelled by human observers were considered for detection. The results of our automated method (Fig. 4d) and the reference standard (Fig. 4b) are very similar, whether images contained many or few abnormal vessels. In contrast, the second human observer appears to over-detect abnormal vessels.

## Discussion and Conclusions

We have developed a framework for automated detection of three types of leakage (large focal, punctate focal, and vessel segment), and have tested it on FA images from patients with malarial retinopathy. The framework benefits from three major components: vessel segmentation, saliency map generation, and leakage detection. The results of our framework are comparable to dual grading with consensus by expert human observers. Our method demonstrated satisfactory overall performance on large focal, punctate focal and vessel segment leakage detection, with sensitivities of 0.95, 0.82, and 0.81, respectively.

Automated analysis of retinal images is an important objective in medical research. Traditionally the main emphasis has been on automated analysis of colour fundus photographs rather than FA images, and on quantifying vessel geometry rather than identifying particular vessel segments or non-vessel regions affected by focal lesions. As a result the problem of detecting leakage is relatively unexplored. To the best of our knowledge this is the first report of automated analysis of medical images to detect multiple distinct types of leakage.

Achieving high accuracy in automated medical image processing is challenging, and several different factors can compromise performance. First of all, the appearance of features of interest can vary hugely depending on the condition of the patient. Children with cerebral malaria may have nystagmus, roving eye movements, and corneal dryness due to incomplete lid closure. These issues can lead to poor image quality. There is often a very large variation in brightness, contrast, and artefact across images; and this makes it difficult to have universal criteria to define the leaking area. Secondly, punctate and vessel segment leakage can be difficult to grade, even for expert human graders. It is possible that an automated technique such as ours might provide more accurate detection than the current human expert reference standard.

Reasons for the lower sensitivity in detecting punctate focal leak, compared to large focal leak, are as follows. Some punctate focal leak areas close to vessels were falsely determined as vessel segments and were removed by step 4 of the automated process to detect punctate focal leakage. Other sites of punctate leak were very close to each other, and our method detected them as one site rather than several sites.

Another highlight of our method is that the novel adaptation of the concept of salient features to the field of medical image analysis. From a psychological perspective, saliency is a predictor of visual object regions that draw more attention to human. It illustrates the relative importance of constituents of our visual world, and is closely involved in perception and processing of visual stimuli. Computationally, saliency refers to a region or object that significantly distinct from its surroundings. In this paper, we represented leaking areas as salient regions since they normally stand out from their neighbours in terms of intensity and blur. This feature is in line with the definition of saliency in computer vision field: the salient region is always significantly different from nearby regions in terms of contrast or shapes.

Development of this framework was motivated by medical demands for a tool to measure various types of leakage in patients with CM, and thereby allow better estimation of associations between MR and clinical outcome. However, the flexibility of our framework also suggests it can be extended to detect leakage in other retinal or neurovascular diseases, such as diabetic retinopathy. Therefore we expect our framework to have wider clinical applications.

In conclusion, we have proposed and evaluated an innovative framework to detect three types of leakage, with the aim of supporting the study of malarial retinopathy. Our results suggest our automated method is effective, compared to manual grading by expert observers. These results depend in part on applying the concept of saliency to medical image processing. Our framework has the potential to be developed further as a useful tool for fast, accurate and objective assessment of leak in a range of retinal diseases.

## Method

The proposed automated leakage detection framework consists of three steps: vessel segmentation, saliency generation, and leakage detection. The detection framework in three types of leakage was implemented in Matlab. All the experiments were performed on a PC with 3.1 GHz Intel Core system and 8 GB RAM. In the following subsections, these steps will be introduced.

### Vessel Segmentation

The automated detection of blood vessels is a prerequisite in the development of automated system for the analysis of leakage. For this work, we adopted a state-of-the-art segmentation technique based on local phase enhancement and graph cut method for its good accuracy and efficiency^21^.

### Local Phase-based Vessel Enhancement

In the method proposed by Zhao *et al.*^21^, local phase enhancement technique is employed to enhance vessel-like structures in an image. As suggested by the name, this filter enhances vessel-like structures by the local phase information in an image instead of the intensity values. As such it is invariant to intensity inhomogeneity within the image and is also capable of producing more reliable results when compared to intensity based filters^22^,^23^,^24^.

Local phase of an image can be estimated by quadrature filters. A quadrature filter comprises a pair of even and odd filters with phase difference of *π*/2. This suggests that image edges align with the zero crossing of the real part of the phase map (the imaginary part will be small). If a line is brighter than the surrounding pixels, the real part of the response gives positive values inside the line and negative values outside. If the line is darker, the values are reversed. In order to enhance all the structures in a given image, multiple scales will be needed. The sum of the weighted responses at each scale is then normalised by the sum of the magnitude of the filter response vectors over all scales. The real part of the normalised sum is used as ‘vesselness map’ (local phase map). This ‘vesselness map’ has some unique properties. It has a positive value inside the lines (or vessels) but a negative value in the background. As designed, it has a zero value at the edge of the line structures. This edge map is processed further to segment the vessels.

### Graph Cut-based Active Contour Method

**Figure 5:**
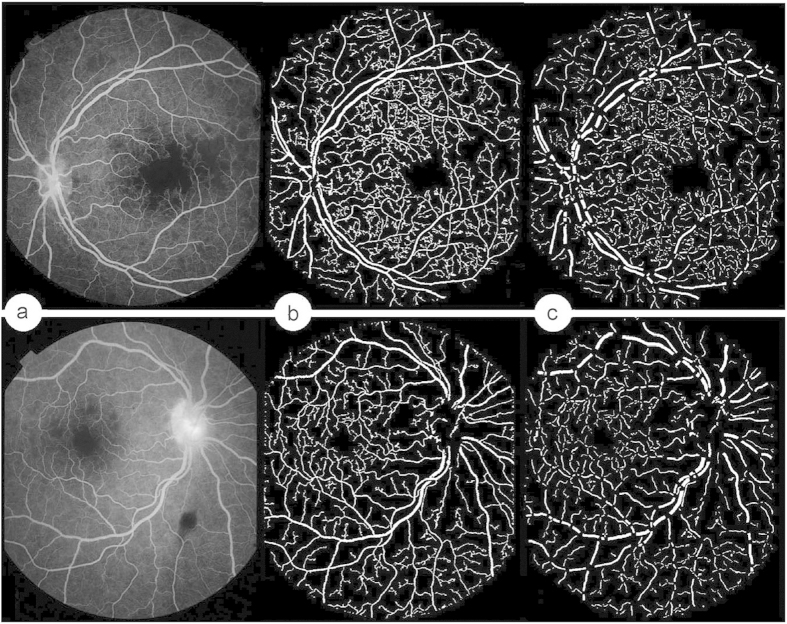
Vessel segmentation. (**a**) Example FA images. (**b**) Segmentation. (**c**) Branch pixels are removed from (**b**).

As proposed by Zhao *et al.*^21^, the retinal vessels are segmented by applying a graph-cut based Chan-Vese (CV) model to the vesselness map^25^. More specifically, let 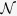 be the set of edges {(*i*, *j*)}, and 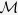 denote the number of image pixels, the discrete energy function can be given as:


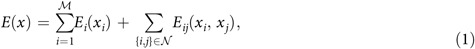


where 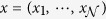 is the binary labelling where the *x*_*i*_ is either 0 or 1 depending on whether the pixel *i* belongs to background or foreground. The unary term *E*_*i*_ and binary term *E*_*ij*_ are defined as:


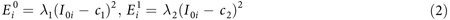



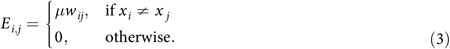


where 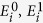 denote the weights between the node *i* and the two terminals while *w*_*ij*_ denotes the weight between neighbouring pixels *i* and *j*. Figure 5a shows the original FA images, and their segmentation results are illustrated on Fig. 5b.

### Geometric Analysis of Vessels

Following the vessel segmentation step, geometrical analysis of the segmented vessels is performed. The morphological thinning algorithm is applied to the segmented vessel trees in order to estimate the centre line and diameter. The exterior pixels from the segmented vessels are removed iteratively by using a thinning algorithm, and obtaining a new binary image containing connected lines of only pixels locating along the vessel centres. A least-squared cubic spline technique is used to obtain smoother trajectories for the centrelines. A centripetal scheme with appropriate parameterizations is employed^26^. Segments with a short centreline (<10 pixels) are removed to improve the speed of later processing.

The vessel diameters are then calculated by using the distance transform of the inverted binary segmented image. It uses the Euclidean distance of each vessel pixel from the closest non-vessel pixel, and thus, doubling the maximum values of the distance transform along the thinned centrelines provides an estimate of the diameter of every vessel segment at its widest point. A centreline is removed if it contains fewer pixels than its estimated diameter.

In addition, the branch pixels ( 2 neighbours) are removed, leading to division of the vascular tree into individual vessel segments. This is useful for diameter analysis, since diameters at branch locations are hard to define. Figure 5c demonstrates the vessel segments after removing the branch pixels of Fig. 5b.

### Saliency Generation

Let *r*(*k*) to be the viable local representation as a patch that represents pixel *k* (here, a 3 × 3 window centred on pixel *k* is used to define the patch). Therefore, the saliency value of a patch *r*(*k*) can be defined as


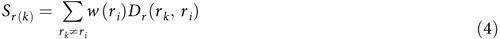


where *w*(*r*_*i*_) is the weight of region *r*_*i*_. Here we use the mean of the intensity values of patch *r*_*i*_ as *w*(*r*_*i*_). *D*_*r*_(*r*_*k*_, *r*_*i*_) measures the distance of the intensity between two regions *r*_*k*_ and *r*_*i*_:


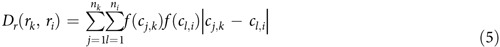


where *n*_*k*_ denotes the number of pixel in region *r*_*k*_, *c*_*j*,*k*_ indicates the *j*^*th*^ intensity value among all *n*_*k*_ relative intensity values in the region *r*_*k*_, and *f*(*c*_*j*,*k*_) is the frequency of the intensity value *c*_*j*,*k*_, estimated by building a histogram over all *c*_*j*,*k*_ values of pixels in the selected patch. The number of bins in the histogram was set as 25.

We incorporated spatial information by applying a weighting term into Equation (4) to increase the effects of closer regions and decrease the effects of more distant regions. Specifically, for any region *r*_*k*_, the spatially saliency value is defined as:


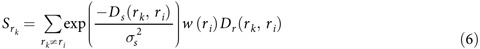


where *D*_*s*_(*r*_*k*_, *r*_*i*_) is the spatial distance between patches *r*_*k*_ and *r*_*i*_. The spatial distance between two patches is defined as the Euclidean distance between the centroids of the respective patches. *σ*_*s*_ controls the strength of spatial weighting. Larger values of *σ*_*s*_ increase the effect of spatial weighting, so that more distant segments would contribute more to the saliency estimation of the current patch. In our implementation, we set 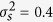. In our application, the intensity-based saliency finally will be normalised into range0,1.

### Large Focal Leakage Detection

A 6-step process was used to detect large focal leakage.

**Figure 6:**
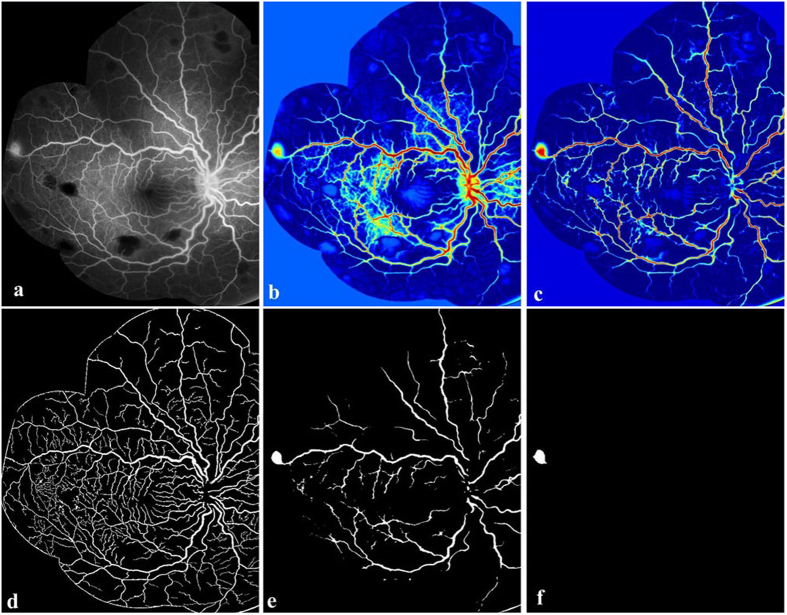
Overview of the main steps taken by our algorithm for detecting large focal leakage. (**a**) Example FA image. (**b**) Saliency map of (**a**). The warmer colour indicates the more salient regions, and the cooler colour shows the less salient regions, and the appearance of this leak is highlighted compared to the original image. (**c**) Retinex applied to (**b**). (**d**) Vessel segmentation results. (**e**) Binary image of (**c**). (**f**) The detected large focal leakage region.

1. Estimation of the saliency map from the input FA image (as proposed in Section 2). Normalize the saliency map into range of 0 − 1. Figure 6b shows the saliency map of input FA image (Fig. 6a). The large focal leaking area, optic disc and vessels have been highlighted with warm colour (salient region).
2. Correction of inhomogeneity in the saliency map. We used Retinex (for more details see^21^). The Retinex theory originally dealt with colour constancy, which in human vision ensures that the perceived colours of objects remain relatively constant under varying illumination conditions. We used Retinex to address the related problem of heterogeneous brightness. Figure 6c demonstrates the saliency map after the applying Retinex on Fig. 6b. Retinex successfully corrects the contrast between the vessels and background.
3. Determination of a binary image from the output of step 2. Most of the image background has been removed, and the large focal leaking area and large vessels are retained, as shown on Fig. 6e.
4. Masking of vessel segmentation results (Fig. 6d) on the output of step 3. All the vessel regions will be replaced by ‘0’ (black).
5. Removing the small regions retained after step 4 applied. For each image, we have prior knowledge on the diameter of the optic disc, which were measured by our graders. As the definition of large focal leakage, the greatest linear diameter of the leaking region is 125 *μ*m. The major axis lengths of the retained regions are computed and any segments with a major axis length of ≤ 125 *μ*m will be removed.
6. Removal of the optic disc. For some cases, the optic disc is retained after step 5. The optic disc can be located by counting the number of nearby vessels of the retained regions. Normally, the number of vessels surround the optic disc is much larger than the number of vessels close to large focal leaking sites. In this study, the threshold number is empirically chosen as 5. In other words, if a region is remained after step 5, and its surrounding vessel number is larger than 5, this region will be assumed as the optic disc, and will be removed. Figure 6f indicates the retained region after the above steps, and this region is defined as the large focal leaking region.

### Punctate Focal Leakage Detection

**Figure 7:**
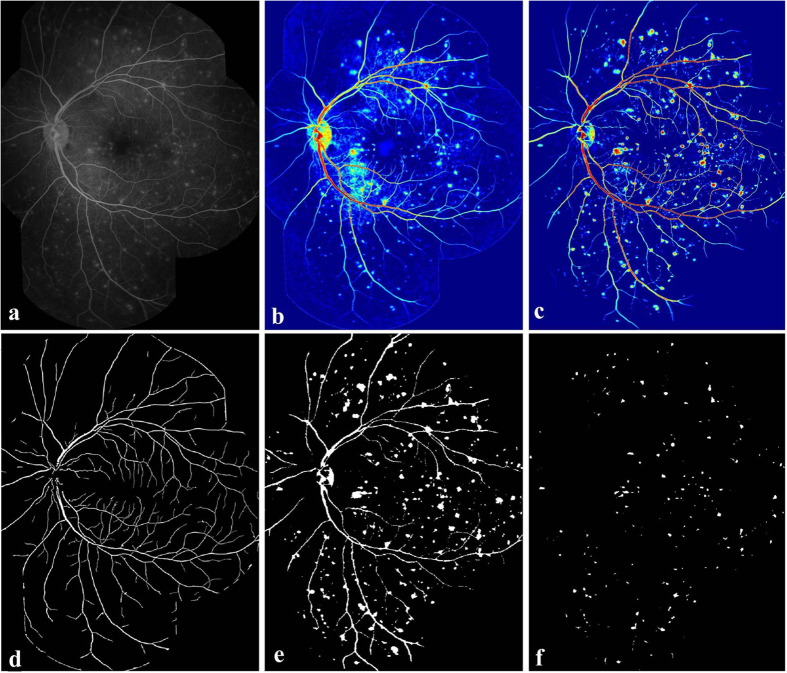
Overview of the main steps taken by our algorithm for detecting punctate focal leakage. (**a**) Example FA image. (**b**) Saliency map of (**a**). (**c**) Retinex applied to (**b**). (**d**) Vessel segmentation results. (**e**) Binary image of (**c**). (**f**) The detected punctate focal leakage regions.

1. Repeat step 1− 4 of large focal leakage detection. The result after each step is shown in Fig. 7a–e.
2. Remove the large regions retained after step 4 applied. As we defined the leaking regions which the greatest linear diameter are <125 *μ*m as punctate leaking regions, any retained segments with a major axis length of ≥125 *μ*m will be removed (the optic disc also is equally eliminated). Figure 7f indicates the detected punctate focal leaking regions. The centroid of these regions is defined as the centre point of the punctate focal leakage.

### Vessel Segment Leakage Detection

The leaking vessels can be detected in a similar way to those for the two other types of leakages.

**Figure 8:**
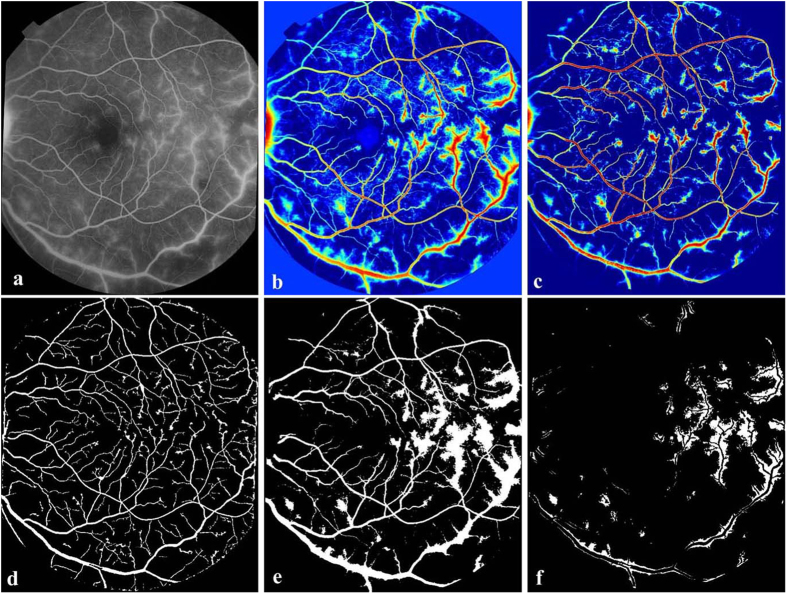
Overview of the main steps taken by our algorithm when detecting leaking vessels. (**a**) Example FA image. (**b**) Saliency map of (**a**). (**c**) Retinex applied to (**b**). (**d**) Vessel segmentation results. (**e**) Binary image of (**c**). (**f**) The detected leaking areas from their closest vessels.

1. Repeat step 1− 4 of large focal leakage detection. The result of each step of the proposed vessel segment detection is shown in Fig. 8a–e.
2. The retained regions are the leaking areas from their closest vessels. The region size which is smaller than 10 pixels will be eliminated. Based on the location of the retained leaking regions shown as Fig. 8f and the vessel segmentation results, the leaking vessels can be located easily.
3. Repeat step 6 of large focal leakage detection to remove the optic disc.

## Additional Information

**How to cite this article**: Zhao, Y. *et al.* Automated Detection of Leakage in Fluorescein Angiography Images with Application to Malarial Retinopathy. *Sci. Rep.* **5**, 10425; doi: 10.1038/srep10425 (2015).

## Figures and Tables

**Figure 1:**
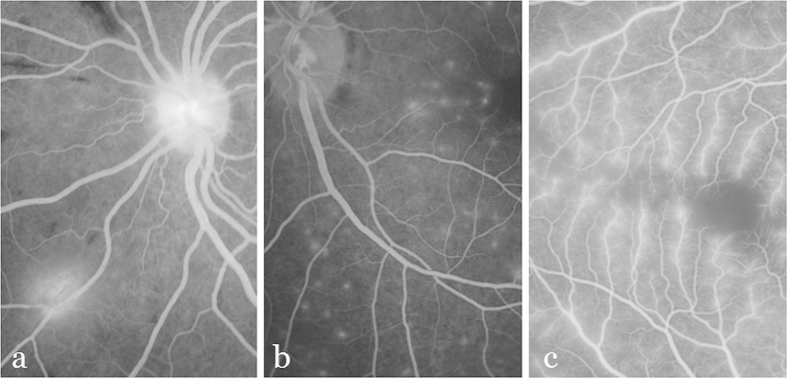
Illustration of three types of leakage. (**a**) Large focal leakage. (**b**) Punctate leakage. (**c**) Vessel leakage.

**Table 1:** The performance of the proposed framework to detect large focal and punctate focal leakage.

	**large focal**	**punctate focal**
sensitivity of leakage detection	0.95	0.82
false positive per image	0	1.3
false negative per image	0.1	2.6
ratio of overlapping area	0.89	-

**Table 2:** The performance of the proposed framework to detect vessel segment leakage.

	**sensitivity**	**specificity**	**accuracy**	**AUC**
second observer (DGP)	0.793	0.736	0.758	0.765
proposed method	0.808	0.821	0.804	0.815
